# Enhancing long-term knowledge retention in undergraduate stroke education through virtual simulation: a randomized study

**DOI:** 10.3389/fmed.2026.1878200

**Published:** 2026-07-29

**Authors:** Xiaohua Shi, Chunpeng Xu, Hongyu Liu, Yang Cui, Zhongyu Zhao, Zhongxin Xu, Jing Mang

**Affiliations:** 1 Department of Neurology, China-Japan Union Hospital of Jilin University, Changchun, China; 2 Jilin Provincial Clinical Medicine Research Center of Behavioral Neurology, Changchun, China

**Keywords:** knowledge retention, medical education, simulation, stroke, teaching

## Abstract

**Background:**

Virtual simulation is increasingly used in undergraduate medical education, but its value for long-term retention in stroke teaching remains unclear. This trial compared virtual simulation-based teaching with conventional bedside teaching in undergraduate stroke education.

**Methods:**

This was a single-center, prospective, parallel-group, randomized educational trial with a 1:1 allocation ratio. Fourth-year undergraduate medical students were assigned to a simulation group or a conventional group. Both groups received the same didactic teaching followed by practical training in different formats with equal teaching hours. Outcomes were assessed 1 day before the module, 1 day after the module, and 10 months later. The primary outcome was the 10-months follow-up assessment score. Secondary outcomes included the post-course assessment score, decay score, and score trajectories over time.

**Results:**

A total of 110 students were randomized, with 55 assigned to each group; 107 were included in the final longitudinal analysis. At the 10-months follow-up, assessment scores were higher in the simulation group than in the conventional group (median [IQR], 81.0 [77.0–85.5] vs. 74.5 [71.0–78.0]). After adjustment for pre-course assessment score and prior academic performance, the simulation group remained associated with higher follow-up scores (adjusted mean, 81.18 vs. 74.81; adjusted mean difference, 6.37 points; 95% CI, 4.83–7.91; *P* < 0.001). The primary result remained stable after additional adjustment for self-reported re-exposure during follow-up (adjusted mean difference, 5.92 points; 95% CI, 4.35–7.49; *P* < 0.001). Post-course scores were also higher and decay scores were lower in the simulation group. No adverse or unintended events were identified.

**Conclusion:**

Virtual simulation-based teaching was associated with higher immediate post-course scores and better long-term retention in undergraduate stroke education, supporting its use as a valuable adjunct to conventional bedside teaching.

## 1 Introduction

Stroke is a core component of undergraduate neurology education. Consequently, effective stroke teaching requires students not only to master core clinical concepts but also to integrate key competencies within dynamic clinical contexts, including the accurate recognition of evolving neurological deficits, the interpretation of diagnostic findings, and the formulation of timely management strategies. These demands arise within a discipline that many learners perceive as difficult, and they make stroke particularly challenging to teach through routine clinical exposure alone (1, 2).

Conventional stroke teaching usually combines didactic instruction with traditional supervised bedside teaching. This approach remains valuable because it allows students to encounter real patients and observe clinical practice directly (2). However, its educational yield may be constrained by limited case availability, heterogeneity of presentation, time pressure in acute care settings, and the need to prioritize patient safety and clinical service demands (2, 3). In addition, students often participate passively during bedside teaching and may have limited opportunities to rehearse assessment and decision-making in a structured and repeatable way. Similar limitations of bedside-based neurology teaching, including reduced active engagement and restricted opportunities for standardized practice, have also been noted in previous studies (2, 4).

Virtual simulation has emerged as a promising adjunct in medical education because it can provide standardized, interactive, and reproducible learning experiences (3, 5). By recreating clinical scenarios in a controlled environment, virtual simulation may help learners apply knowledge in context, strengthen clinical reasoning, and engage more actively in the learning process (3, 5). In stroke teaching, this approach may be particularly useful because it allows students to work through typical and evolving presentations, practice key steps in assessment and initial management, and revisit critical decision points without placing additional demands on patients or clinical services (6). Prior work in medical and neurology education has suggested that simulation-based teaching is feasible, well accepted by learners, and particularly suitable for acute neurological conditions such as stroke, in which timely recognition, decision-making, and teamwork are essential (3, 5–9).

However, much of the existing evidence has relied on learner perception, preference, confidence, or short-term outcomes rather than direct comparison of objective educational outcomes (4, 7–10). This issue is especially relevant in stroke teaching because suspected acute stroke is often first evaluated in emergency or other non-specialist settings, where delayed or missed recognition can occur, and because early assessment and initial management are highly time-sensitive (11, 12). For this reason, the value of a teaching strategy in undergraduate stroke education should not be judged solely by immediate post-course performance but also by whether clinically relevant knowledge remains accessible over time. Evidence in the stroke section of undergraduate neurology education remains limited, particularly regarding whether the educational advantages of virtual simulation extend to longer-term retention of stroke-related knowledge when compared with traditional supervised bedside teaching. Therefore, the present study compared didactic teaching combined with traditional supervised bedside teaching with didactic teaching combined with virtual simulation in order to evaluate the educational value of virtual simulation in undergraduate stroke teaching.

## 2 Materials and methods

### 2.1 Study design and participants

This was a single-center, prospective, parallel-group randomized educational study conducted in the stroke module of the undergraduate neurology course at the China-Japan Union Hospital of Jilin University during the autumn semester of 2023. The study was approved by the Ethics Committee of China-Japan Union Hospital of Jilin University (No. 20221208016), and written informed consent was obtained from all participants before enrollment.

Eligible participants were fourth-year undergraduate medical students enrolled in the stroke module. Students were included if they were aged 18 years or older, attended the scheduled teaching activities, and were expected to complete the pre-course, post-course, and 10-months follow-up assessments. Students who did not complete the required assessments were excluded from the final analysis. Baseline data collected before randomization included age, sex, prior academic performance, and pre-course assessment score. Prior academic performance was defined as the cumulative academic average before the start of the module. This trial is reported in accordance with the CONSORT 2025 statement for randomized trials (13).

### 2.2 Interventions and randomization

Participants were assigned in a 1:1 ratio to the conventional teaching group or the simulation group using a block randomization sequence generated by an independent researcher who was not involved in teaching, outcome assessment, or data analysis (Hongyu Liu). Allocation was concealed using sequentially numbered, opaque, sealed envelopes, which were opened only after enrollment.

The stroke module was delivered as part of the undergraduate neurology curriculum and consisted of a didactic component and a practical component. All students first received the same didactic teaching, which comprised 10 teaching periods of 45 min each (total, 450 min). This component provided the theoretical foundation of the module and covered the core domains of undergraduate stroke education.

Following the didactic component, students underwent practical teaching in one of two formats, with equal teaching hours in the two groups. The conventional group received supervised bedside teaching in the ward setting, including bedside observation, instructor-led demonstration, and case-based discussion (Zhongxin Xu and Yang Cui). Teaching was organized according to the routine clinical teaching schedule and the availability of appropriate cases, while remaining aligned with the predefined objectives of the stroke module. The simulation group received virtual simulation-based training organized into nine structured sections, each comprising two teaching periods, for a total of 18 teaching periods (Xiaohua Shi and Chunpeng Xu). The curriculum was built around progressive stroke-related scenarios, and each section followed a standardized format of briefing, scenario-based virtual practice, debriefing and feedback, and knowledge consolidation or a short quiz (Table 1).

**TABLE 1:** Structure of the virtual simulation curriculum.

Section	Topic	Briefing and objectives	Scenario-based practice	Debriefing and feedback	Consolidation
1	Hyperacute stroke recognition and emergency response	Warning signs and emergency workflow	Recognition of acute stroke and activation of the emergency pathway	Recognition delays and key decision points	Key points in acute stroke recognition
2	Focused neurological assessment	Examination goals and key neurological signs	Focused neurological examination and deficit interpretation	Examination sequence and localization reasoning	Key examination and localization points
3	Neuroimaging interpretation	Key imaging features in stroke evaluation	Interpretation of CT/MRI and vascular imaging findings	Imaging pitfalls and links to treatment decisions	Key imaging interpretation points
4	Intravenous thrombolysis	Indications, contraindications, and treatment goals	Eligibility assessment and initial thrombolysis decision-making	Eligibility pitfalls and safety considerations	Key thrombolysis decision points
5	Mechanical thrombectomy and reperfusion strategy	Reperfusion strategy and thrombectomy decision points	Reperfusion selection and angiographic scenario interpretation	Thrombectomy decisions and IPAS-related angiographic signs	Key reperfusion strategy points
6	Intracerebral hemorrhage	Recognition and early management priorities	Identification of intracerebral hemorrhage and initial management planning	Diagnostic pitfalls and early management priorities	Key hemorrhagic stroke management points
7	Aneurysmal subarachnoid hemorrhage	Typical presentation and diagnostic pathway	Evaluation of suspected aneurysmal subarachnoid hemorrhage	Diagnostic pathway and initial management	Key subarachnoid hemorrhage recognition and diagnostic steps
8	Stroke mimics and differential diagnosis	Common mimics and differentiation strategy	Differentiation between stroke and mimic presentations	Diagnostic pitfalls in differential diagnosis	Key differential diagnosis points
9	Integrated stroke management with multidisciplinary collaboration	Integrated management and team-based goals	Complete stroke scenario from recognition to treatment planning	Clinical reasoning and communication points	Integrated case review

Each section comprised two teaching periods and followed the same time structure: 10 min of briefing and objectives, 45 min of scenario-based virtual practice, 25 min of debriefing and feedback, and 10 min of knowledge consolidation or a short quiz. CT, computed tomography; MRI, magnetic resonance imaging; IPAS, intraprocedural angiographic signs.

Both practical formats were designed to address the same core learning objectives, namely early recognition of acute stroke, focused neurological examination, interpretation of imaging findings, formulation of initial treatment strategies, and clinical reasoning in time-sensitive neurological situations. In the simulation curriculum, selected thrombectomy-related angiographic scenarios drew in part on the concept of intraprocedural angiographic signs (IPAS) (14). Written assessments were organized and scored separately (Jing Mang), and statistical analysis was performed independently (Zhongyu Zhao).

### 2.3 Outcome assessment

Educational outcomes were assessed at three prespecified time points: 1 day before the start of the module, 1 day after completion of the module, and 10 months after completion of the module. Three parallel written test forms were developed to minimize testing effects related to repeated exposure to identical items. Each test form consisted of 24 questions with a total score of 100 points, including 10 A1-type single-best-answer questions worth 3 points each, 10 A2-type single-best-answer questions worth 3 points each, and 4 case analysis questions worth 10 points each. Together, these items assessed direct knowledge application, case-based clinical reasoning and decision-making, and more integrative clinical judgment. All three forms were constructed according to the same test blueprint, covered the same core learning objectives of the stroke module, and were developed and reviewed by three senior neurologists before use.

The primary outcome was the total score on the 10-months follow-up assessment, which was prespecified as the main indicator of long-term retention. Secondary outcomes included the post-course assessment score, score changes across the three assessment time points, and a decay score calculated as the post-course score minus the 10-months follow-up score, with lower values indicating better long-term retention. At the 10-months follow-up assessment, students self-reported whether they had any re-exposure to neurology-related content after the post-course assessment. Detailed information on the mode, timing, and frequency of re-exposure was not collected.

Harms or unintended events were defined as any study-related event that could adversely affect participants’ well-being, privacy, academic participation, or ability to complete the study, and were monitored through instructor observation and participant self-report during the teaching period and follow-up.

### 2.4 Statistical analysis

No formal sample size calculation was performed because this was a course-based educational study, and all eligible students enrolled in the 2023 autumn stroke module were considered for inclusion. Statistical analyses were conducted on a complete-case basis, and students lost to follow-up were excluded from the final analysis.

Continuous variables were summarized as mean ± standard deviation (SD) or median (interquartile range [IQR]) according to their distribution, and categorical variables as counts and percentages. Baseline characteristics were compared using the independent-samples *t*-test or Mann–Whitney U test for continuous variables, and the chi-square test or Fisher’s exact test for categorical variables, as appropriate. The primary analysis compared 10-months follow-up scores between groups using analysis of covariance, with teaching group as the fixed factor and pre-course assessment score and prior academic performance as covariates. A sensitivity analysis additionally adjusted the primary model for self-reported re-exposure to neurology-related content during follow-up. To assess knowledge retention after course completion, decay scores were compared between groups using a general linear model adjusted for post-course score. A further model additionally adjusted for prior academic performance and self-reported re-exposure during follow-up. As a supplementary longitudinal analysis, changes in scores across the three assessment time points were examined using a mixed-effects model for repeated measures, with group, time, and the group-by-time interaction as fixed effects and participant as a random effect. Internal consistency of the assessment forms was evaluated using Cronbach’s alpha, and item-level performance was further described using item difficulty indices, discrimination indices, and corrected item-total correlations. Effect sizes were reported as Cohen’s d for observed between-group differences and partial η^2^ for the adjusted primary ANCOVA model. Data analysis was performed independently by designated personnel using R (version 4.1.3), and a two-sided *P*-value < 0.05 was considered statistically significant.

## 3 Results

### 3.1 Participant flow and baseline characteristics

**FIGURE 1:**
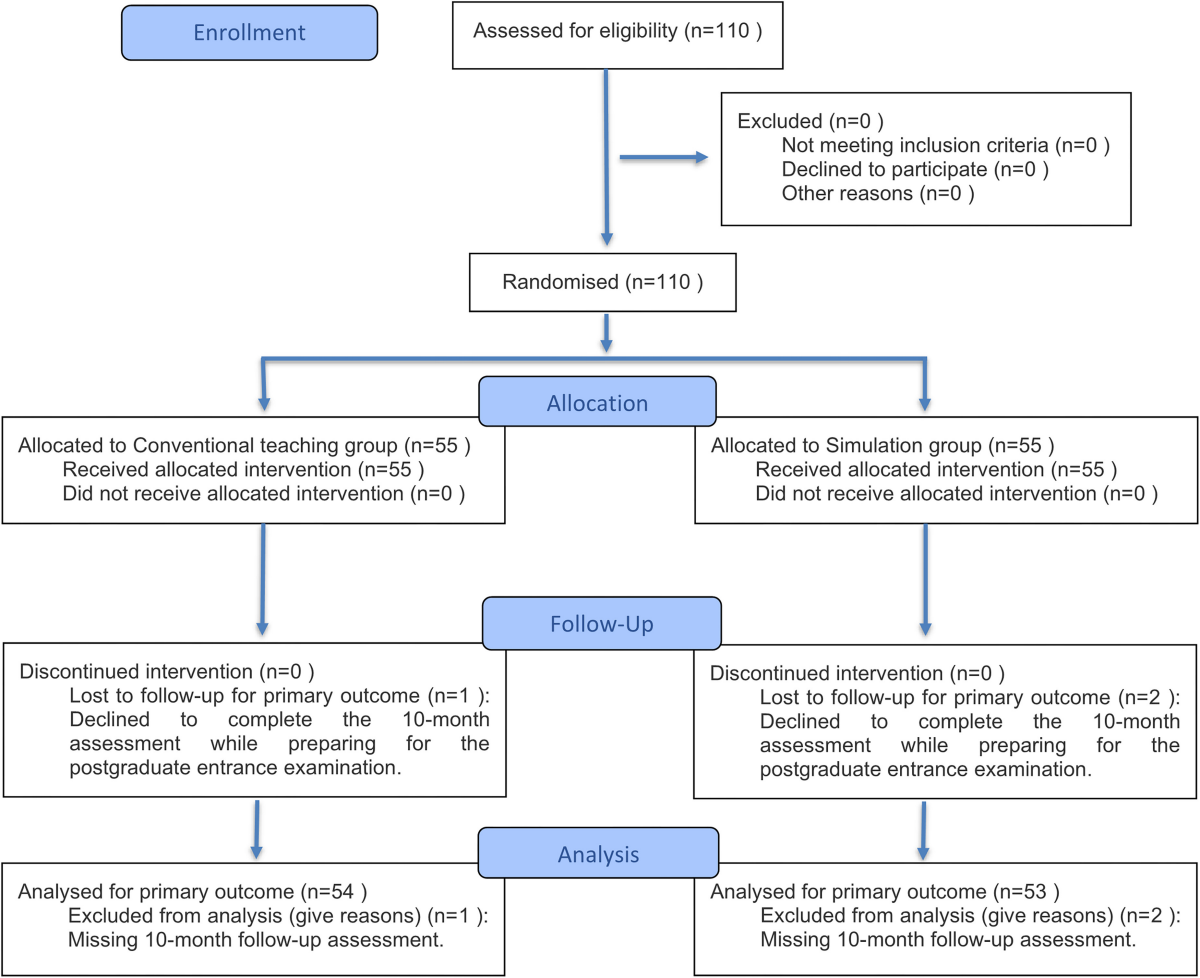
CONSORT 2025 flow diagram of participant enrollment, allocation, follow-up, and analysis.

A total of 110 fourth-year undergraduate medical students were assessed for eligibility and randomized in a 1:1 ratio to the simulation group (*n* = 55) or the conventional group (*n* = 55). All randomized students received the allocated intervention and completed the post-course assessment. At the 10-months follow-up, 107 students completed the long-term assessment and were included in the final longitudinal analysis, including 53 students in the simulation group and 54 in the conventional group; loss to follow-up occurred in 2 students in the simulation group and 1 in the conventional group because they declined further participation while preparing for the postgraduate entrance examination (Figure 1).

The two groups were well balanced at baseline, with no significant between-group differences in age (22 [21–23] vs. 22 [21–22], *P* = 0.417), female sex (33/55 [60.0%] vs. 34/55 [61.8%], *P* = 0.845), prior academic performance (73.52 [68.48–79.13] vs. 72.23 [68.00–77.47], *P* = 0.921), or pre-course assessment score (67 [61.25–73] vs. 64 [58–72], *P* = 0.163) (Table 2).

**TABLE 2:** Baseline characteristics.

Variable	Conventional group (*n* = 55)	Simulation group (*n* = 55)	*P*-value
Age, years	22 (21–23)	22 (21–22)	0.417
Female, *n* (%)	33 (60.0)	34 (61.8)	0.845
Prior academic performance	73.52 (68.48–79.13)	72.23 (68.00–77.47)	0.921
Pre-course assessment score	67 (61.25–73)	64 (58–72)	0.163

Cronbach’s alpha coefficients for the pre-course, post-course, and 10-months follow-up assessment forms were 0.772, 0.750, and 0.763, respectively, indicating acceptable internal consistency. Item-level analyses showed mean difficulty indices of 0.579, 0.929, and 0.733 and mean discrimination indices of 0.270, 0.100, and 0.217 for the pre-course, post-course, and follow-up forms, respectively. Corrected item-total correlations were below 0.20 for 11, 19, and 11 items, respectively, although no item showed a negative corrected item-total correlation.

### 3.2 Primary outcome

At the 10-months follow-up, assessment scores were higher in the simulation group than in the conventional group (median [IQR], 81.0 [77.0–85.5] vs. 74.5 [71.0–78.0]; Cohen’s *d* = 0.87). After adjustment for pre-course assessment score and prior academic performance, the simulation group remained associated with higher follow-up scores (adjusted mean, 81.18 vs. 74.81; adjusted mean difference, 6.37 points; 95% CI, 4.83–7.91; *P* < 0.001; partial η^2^ = 0.396). Self-reported re-exposure to neurology-related content during follow-up was reported by 16 of 54 students (29.6%) in the conventional group and 27 of 53 students (50.9%) in the simulation group. This finding remained unchanged after additional adjustment for self-reported re-exposure during follow-up (adjusted mean difference, 5.92 points; 95% CI, 4.35–7.49; *P* < 0.001), and re-exposure was also significantly associated with the follow-up score (*P* = 0.034) (Table 3).

**TABLE 3:** Primary and secondary outcomes.

Outcome	Conventional group	Simulation group	Effect estimate (95% CI)	*P*-value
Primary outcome				
10-months follow-up assessment score, median (IQR) (*n* = 54 vs. *n* = 53)	74.5 (71.0–78.0)	81.0 (77.0–85.5)	–	–
Adjusted 10-months follow-up assessment score*(*n* = 54 vs. *n* = 53)	74.81	81.18	6.37 (4.83–7.91)	<0.001
Sensitivity analysis with additional adjustment for re-exposure†(*n* = 54 vs. *n* = 53)	75.21	81.14	5.92 (4.35–7.49)	<0.001
Secondary outcomes				
Post-course assessment score, median (IQR) (*n* = 55 vs. *n* = 55)	85.0 (82.0–89.0)	88.0 (85.0–91.5)	–	–
Adjusted post-course assessment score*(*n* = 55 vs. *n* = 55)	84.89	87.98	3.08 (1.78–4.38)	<0.001
Decay score, median (IQR) (*n* = 54 vs. *n* = 53)	10.0 (6.0–13.25)	7.0 (3.5–10.0)	–	–
Adjusted decay score‡(*n* = 54 vs. *n* = 53)	9.90	6.99	2.90 (1.24–4.56)	<0.001
Fully adjusted decay score§(*n* = 54 vs. *n* = 53)	9.77	6.66	3.11 (1.60–4.62)	<0.001

Data are presented as median (interquartile range [IQR]) or adjusted mean, as appropriate. For outcomes assessed immediately after the module, analyses were based on 55 students in each group. For the 10-months follow-up and decay score analyses, data were available for 54 students in the conventional group and 53 students in the simulation group. *Adjusted for pre-course assessment score and prior academic performance. †Additionally adjusted for self-reported re-exposure to neurology-related content during follow-up. ‡Adjusted for post-course assessment score. §Further adjusted for post-course assessment score, prior academic performance, and self-reported re-exposure during follow-up.

### 3.3 Secondary outcomes

Immediately after completion of the module, post-course assessment scores were significantly higher in the simulation group than in the conventional group (median [IQR], 88.0 [85.0–91.5] vs. 85.0 [82.0–89.0]; *P* = 0.004; Cohen’s *d* = 0.48). This between-group difference remained significant after adjustment for pre-course assessment score and prior academic performance (adjusted mean, 87.98 vs. 84.89; adjusted mean difference, 3.08 points; 95% CI, 1.78–4.38; *P* < 0.001) (Table 3).

The decline in performance from the post-course assessment to the 10-months follow-up was smaller in the simulation group than in the conventional group (median [IQR] decay score, 7.0 [3.5–10.0] vs. 10.0 [6.0–13.25]; absolute Cohen’s *d* = 0.75). In the unadjusted analysis, the conventional group showed a significantly larger decay score (mean difference, 3.15 points; 95% CI, 1.55–4.75; *P* < 0.001). This finding remained significant after adjustment for post-course assessment score (adjusted mean, 6.99 vs. 9.90; adjusted mean difference, 2.90 points; 95% CI, 1.24–4.56; *P* < 0.001) and after further adjustment for prior academic performance and self-reported re-exposure during follow-up (adjusted mean, 6.66 vs. 9.77; adjusted mean difference, 3.11 points; 95% CI, 1.60–4.62; *P* < 0.001) (Table 3).

**FIGURE 2:**
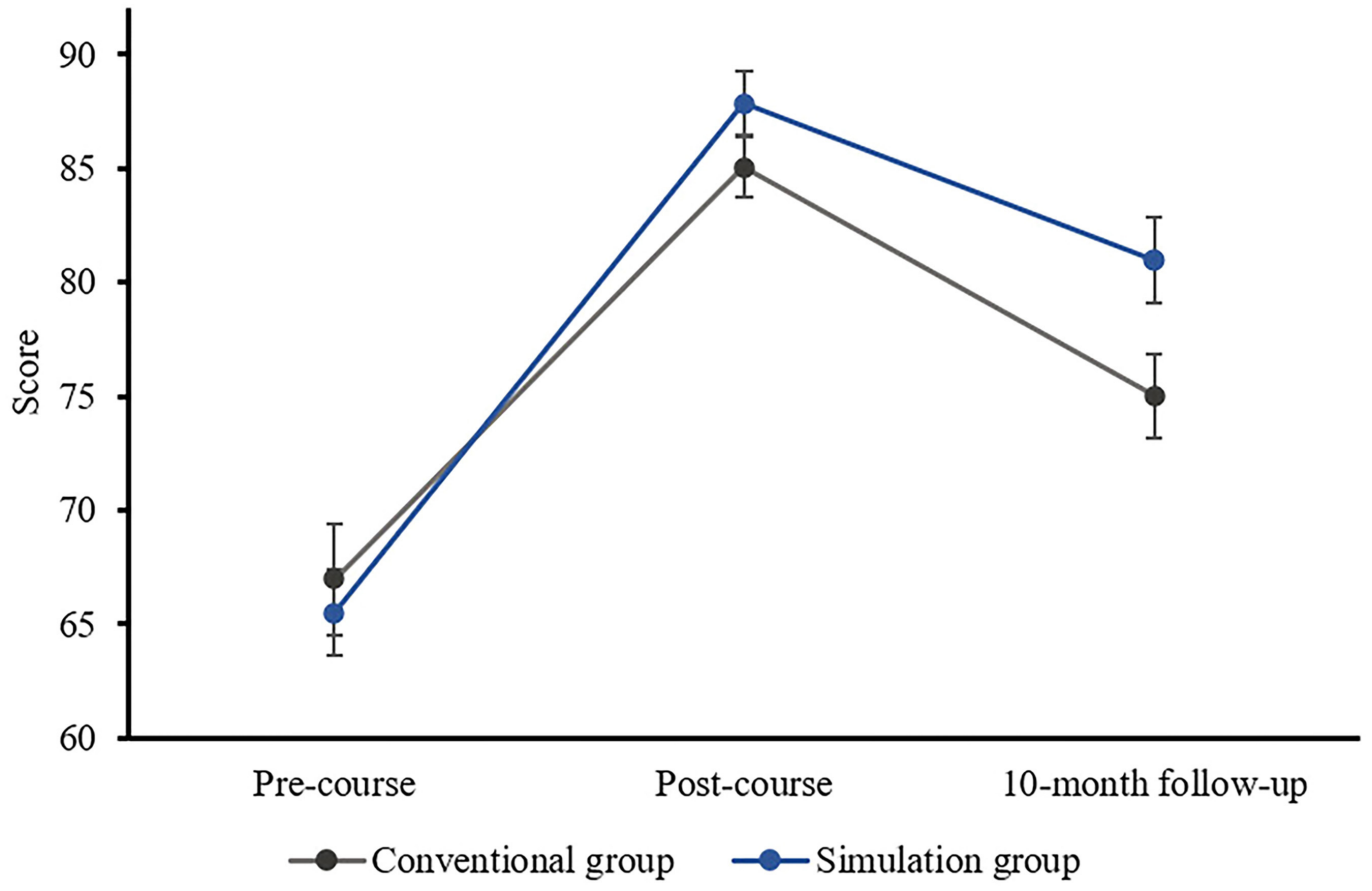
Trajectories of assessment scores across the three study time points in the conventional and simulation groups. Points represent model-estimated means, and error bars indicate 95% confidence intervals. Scores increased in both groups immediately after the module and declined at the 10-months follow-up, but the simulation group maintained higher scores over time and showed a smaller decline from post-course to follow-up.

In the supplementary mixed-effects analysis, there were significant effects of group (*P* = 0.047), time (*P* < 0.001), and group × time interaction (*P* < 0.001), indicating different score trajectories between the two groups. Although scores increased in both groups immediately after the module and declined at 10 months, the simulation group maintained higher scores over time and showed a smaller decline from post-course to follow-up than the conventional group (Figure 2).

### 3.4 Harms and unintended events

No adverse events, unintended events, or withdrawals related to study participation were identified in either group. No confidentiality breaches or study-related complaints were reported during the study period.

## 4 Discussion

In this randomized educational study, students in both groups showed higher scores after completion of the undergraduate stroke module, but the simulation group achieved higher post-course scores and better long-term performance than the conventional group. The adjusted between-group differences were 3.08 points immediately after the module and 6.37 points at 10 months. On a 100-point written assessment, the 10-months difference, together with a large observed standardized difference, suggests a meaningful advantage in the retention of stroke-related knowledge. This advantage should be interpreted as better written knowledge retention rather than direct evidence of improved clinical performance. Overall, these findings suggest that virtual simulation may provide additional educational value beyond the immediate teaching period, particularly for retaining clinically relevant knowledge in time-sensitive areas such as stroke care.

One plausible explanation is that virtual simulation engages learners in a higher level of cognitive activity than bedside observation alone. According to the active–constructive–interactive framework, learning is strengthened when students do not merely receive information passively, but actively generate interpretations, decisions, and responses during task performance (15). In our simulation sessions, students were required to identify symptoms, interpret findings, choose investigations, and make initial management decisions within evolving stroke scenarios. This process may have promoted generative learning, whereby learner-produced responses are retained better than information that is only read or observed, and may also have introduced retrieval practice, which has been associated with improved retention in health professions education (16, 17). The cognitive operations required during simulation also overlapped more closely with those later required in assessment and clinical problem-solving. In addition, the structured debriefing and feedback that followed each simulation may have helped students correct errors, reorganize knowledge, and consolidate more durable task-relevant mental models (6, 18). These features are consistent with broader simulation-based education literature emphasizing the importance of deliberate practice, feedback, curriculum integration, and structured debriefing in effective simulation-based learning (19, 20).

The pattern observed in our study, namely improvement in both groups with greater gains in the simulation group, is broadly consistent with previous work suggesting that interactive and simulation-based methods can enhance stroke-related learning. During the COVID-19 pandemic, interactive virtual video was associated with higher examination scores and greater learner satisfaction than a conventional online classroom in the stroke topic of theoretical neurology, highlighting the educational value of interactivity in stroke teaching (8). In neurology clerkship teaching, simulation-based learning was also rated more favorably than modified bedside demonstration and WeChat-assisted teaching, particularly in relation to diagnostic skills, physical examination, communication, and interest in neurology, although that study mainly relied on subjective evaluations (4). Our findings extend this line of work by incorporating objective assessment across multiple time points rather than relying primarily on learner preference or immediate post-session impressions.

The long-term findings are particularly relevant in stroke education. Acute neurological symptoms are often first encountered by non-neurologists, and early recognition and initial management are highly time-sensitive (11). For this reason, the value of a teaching strategy in undergraduate stroke education should not be judged solely by immediate post-course performance, but also by whether clinically relevant knowledge remains accessible over time. Previous studies have generally shown that virtual or simulation-based approaches are feasible, well accepted, and associated with improved learner engagement, confidence, or short-term educational outcomes, but evidence on more sustained effects has remained limited (6, 9). From this perspective, our long-term assessment suggests that virtual simulation may be especially valuable in stroke teaching, where durable retention of clinically relevant knowledge is essential for early recognition and initial decision-making.

Our findings should not be interpreted as diminishing the educational value of traditional supervised bedside teaching. Bedside teaching remains essential for exposure to real patients, authentic communication, and the realities of clinical workflow (2). Indeed, prior studies have emphasized that simulation should be viewed as a supplement rather than a replacement for bedside-based learning (3). Rather, the present results suggest that virtual simulation may address some of the inherent limitations of bedside teaching in stroke education, particularly the variability of case availability, the limited opportunity for repeated practice, and the difficulty of ensuring that all students encounter comparable acute scenarios. In this sense, virtual simulation may serve as a useful complementary strategy that standardizes key learning experiences while preserving the strengths of real clinical exposure.

Another notable finding was that the primary result remained stable after additional adjustment for self-reported re-exposure to neurology-related content during follow-up, with an adjusted mean difference of 5.92 points at 10 months. This suggests that the observed long-term advantage of simulation was not solely explained by subsequent exposure, although such experiences may still have contributed to overall knowledge maintenance. However, because re-exposure was recorded only as a simplified self-reported indicator, without detailed information on its mode, timing, or frequency, residual confounding related to subsequent learning experiences cannot be completely excluded.

This study has several strengths. First, it focused on a clearly defined and clinically important topic within undergraduate neurology education. Second, both groups received the same didactic teaching, allowing the comparison to focus specifically on differences in practical teaching format. Most importantly, the present study evaluated learning retention over time rather than limiting evaluation to immediate post-course performance. This is particularly relevant in stroke education, where early recognition and initial management are highly time-sensitive and where clinically relevant knowledge needs to remain accessible beyond the teaching period. In this respect, the study adds to the existing simulation literature in neurology and stroke education.

Several limitations should be acknowledged. First, this was a single-center course-based educational study with a modest sample size, which may limit generalizability. No formal pilot study or *a priori* sample size calculation was performed because all eligible students enrolled in the 2023 autumn stroke module were considered for inclusion. Therefore, the study may have been underpowered to detect smaller but educationally meaningful differences. Second, practical teaching in the conventional group depended on routine bedside teaching and case availability, which may have introduced variation in clinical exposure. Third, validity evidence for the written assessment forms was limited. Although the forms were developed according to a shared test blueprint, reviewed by senior neurologists, and examined using internal consistency, item difficulty, discrimination indices, and corrected item-total correlations, item-level analyses suggested variable performance, particularly a possible ceiling effect in the post-course form. Formal content validity indices such as S-CVI or I-CVI were not available. Fourth, re-exposure during follow-up was recorded only as a simplified self-reported indicator without detailed information on its mode, timing, or frequency, so residual confounding related to subsequent learning experiences cannot be completely excluded. Finally, outcomes were based on written assessments rather than observed clinical performance, practical skills, or patient-care outcomes. Future studies should include observed clinical assessments, such as OSCEs, simulation performance ratings, bedside skill assessments, or patient-care proxy outcomes, to determine whether the observed knowledge-retention advantage translates into improved clinical performance.

## 5 Conclusion

In summary, virtual simulation-based training was associated with better long-term retention of stroke-related knowledge than traditional supervised bedside teaching in the undergraduate stroke module. These findings support the use of virtual simulation as a valuable adjunct to conventional bedside teaching, particularly for helping students retain clinically relevant knowledge in time-sensitive areas such as stroke care.

## Data Availability

The raw data supporting the conclusions of this article will be made available by the authors, without undue reservation.
